# Decontamination of medical devices from pathological amyloid-β-, tau- and α-synuclein aggregates

**DOI:** 10.1186/s40478-014-0151-5

**Published:** 2014-10-25

**Authors:** Achim Thomzig, Katja Wagenführ, Martin L Daus, Marion Joncic, Walter J Schulz-Schaeffer, Marc Thanheiser, Martin Mielke, Michael Beekes

**Affiliations:** Prion and Prionoid Research Unit, ZBS 6 – Proteomics and Spectroscopy, Centre for Biological Threats and Special Pathogens, Robert Koch-Institut, Nordufer 20, 13353 Berlin, Germany; Department of Neuropathology, Prion and Dementia Research Unit, University Medical Centre Göttingen, Robert-Koch-Str. 40, 37075 Göttingen, Germany; Department of Infectious Diseases, FG 14 - Applied Infection Control and Hospital Hygiene, Robert Koch-Institut, Nordufer 20, 13353 Berlin, Germany; Department of Infectious Diseases, Robert Koch-Institut, Nordufer 20, 13353 Berlin, Germany

**Keywords:** Alzheimer’s disease, Parkinson’s disease, Dementia with Lewy bodies, Amyloid-β, Tau, α-synuclein, Prion, Aggregate, Decontamination, Medical devices

## Prion-like features and possible hazards of amyloid-β-, tau- and α-synuclein aggregates

Increasing evidence suggests that the misfolding and aggregation of different disease-associated proteins such as amyloid-β (Aβ) and tau in Alzheimer’s disease (AD), α-synuclein in Parkinson’s Disease (PD) and dementia with Lewy bodies (DLB), or prion protein (PrP) in prion diseases is based on a common molecular mechanism of nucleation-dependent protein polymerization [1-3]. Consistent with this concept it has been recently demonstrated that the aggregation and deposition of Aβ, tau, and α-synuclein in the brain can be stimulated in animal models by injection of inocula that contain aggregated forms of these proteins (for a review see: [4]). Additionally, intracerebral implantation of stainless steel wires previously contaminated with Aβ-containing brain extract was found to stimulate cerebral beta-amyloidosis in APP23 transgenic mice [5], and Aβ aggregates resisted inactivation of nucleating (“seeding”) activity by boiling [5] or formaldehyde [6]. Taken together, these findings raised concerns that the transmission of pathological protein particles from common neurodegenerative diseases may possibly pose a risk to patient safety, e.g. in transfusion medicine or surgery. However, so far neither experimental nor epidemiological studies provided evidence for a transmission of severe or even fatal disease by Aβ-, tau- or α-synuclein aggregates [4]. Yet, stimulation of cerebral protein aggregation by iatrogenically transmitted Aβ-, tau- or α-synuclein particles could possibly have harmful effects below full-blown disease transmission. For α-synuclein such scenarios have been experimentally exemplified. Intracerebrally or intramuscularly injected samples containing aggregated human α-synuclein led to both earlier onset of severe motor dysfunction in and premature death of transgenic mice expressing mutated human α-synuclein [7-9]. In addition, intracerebral injection of similar inocula caused neurotoxic effects and neurological impairments in transmission experiments with wild-type mice [10]. Whether those harmful effects can be also caused by transmitted protein particles in humans who express mutated or normal α-synuclein, Aβ or tau is still unknown.

## Testing the depletion of aggregated amyloid-β, tau and α-synuclein in carrier assays

**Figure 1:**
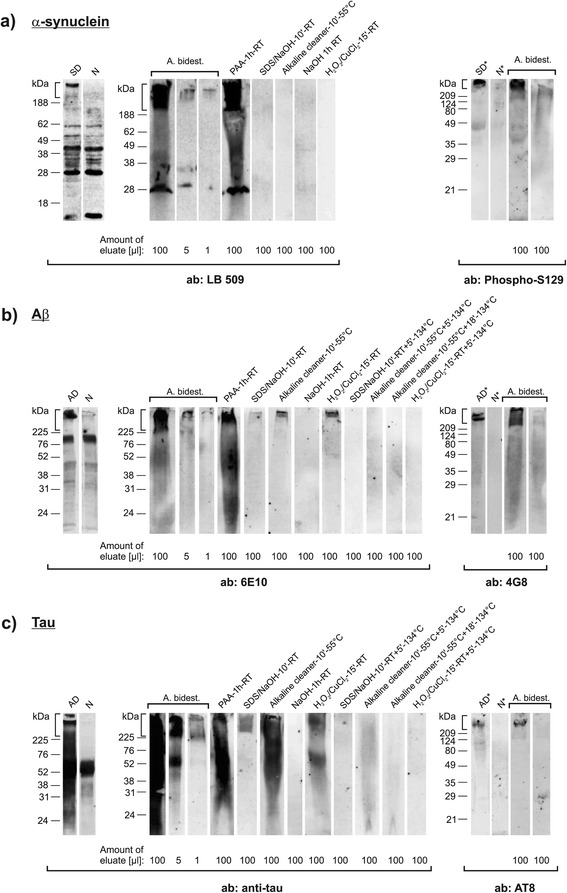
**In vitro carrier assay for testing the depletion of aggregated human α-synuclein, amyloid-β and tau.** Western blot detection of aggregated human α-synuclein **(a)**, Aβ **(b)**, and tau **(c)** by the indicated antibodies (Table 1) in protein eluates from steel wire grids that had been contaminated with 20% (w/v) brain tissue homogenates (BTH) from donors with SD **(a)** or AD (**b** and **c**). Lanes “SD” **(a)**, “AD” (**b** and **c**) and “N” **(a-c)** (“SD*” [a], “AD*” [b and c] and “N*” [a-c]) represent 5 μl (or 20 μl in lanes marked with an asterisk) 20% (w/v) BTH from SD- and AD patients and control donors (without SD or AD), respectively. The identity of α-synuclein, Aβ, and tau aggregates in BTH and on steel wire grids washed with bi-distilled water was confirmed by two different antibodies each (most right lanes in a-c represent steel wire grids that had been contaminated with BTH from control donors). For testing the presence and depletion of aggregated α-synuclein, Aβ, and tau contaminated wires were processed by I) washing with bi-distilled water (a-c, lanes “A. bidest.”), or exposure to II) 0.25% (v/v) peracetic acid for 1 hour at room temperature (RT) (**a-c**, lanes “PAA-1 h-RT”), III) a mixture of 0.2% (w/v) SDS and 0.3% (w/v) NaOH (pH 12.7 - 12.9, non-adjusted) for 10 min. at RT (a-c, lanes “SDS/NaOH-10'-RT”), IV) an alkaline cleaner (0.5%, pH 11.6 - 12.0 [non-adjusted]) [13] for 10 min. at 55°C (**a-c**, lanes “Alkaline cleaner-10'-55°C”), V) 1 M NaOH (pH 13.5 - 13.8, non-adjusted) for 1 h at RT (**a-c**, lanes “NaOH-1 h-RT”), VI) a solution of 7.5% (w/v) H_2_O_2_ containing Cu^2+^ ions [14] (prepared in 100 mM NaCO_3_ [pH 9.5, adjusted] by adding CuCl_2_ to a final concentration of 500 μM) for 15 min. at RT (**a-c**, lanes “ H_2_O_2_/CuCl_2_-15′-RT”), VII) treatment as in III) with subsequent PVSS for 5 min. at 134°C (**b** and **c**, lanes “SDS/NaOH-10′-RT + 5′-134°C”), VIII) treatment as in IV) with subsequent PVSS for 5 min. (**b** and **c**, lanes “Alkaline cleaner-10′-55°C + 5′-134°C”) or 18 min. (**b** and **c**, lanes “Alkaline cleaner-10′-55°C + 18′-134°C”), or IX) treatment as in VI) with subsequent PVSS for 5 min. (**b** and **c**, lanes “H_2_O_2_/CuCl_2_-15′- RT + 5′-134°C”). PVSS was always performed at 3 bar. ab: antibody. Vertical brackets indicate insoluble α-synuclein-, Aβ- and tau aggregates retained in stacking gels.

Thus, the ability to decontaminate medical instruments from aggregated Aβ, tau and α-synuclein may potentially add to patient safety. When discussing this question, data on the efficacy of routinely applicable reprocessing procedures for medical instruments against Aβ-, tau- and α-synuclein aggregates can provide helpful guidance. For this reason, we assessed the activity of different reprocessing procedures against those contaminations in depletion assays that used stainless steel wire grids as surrogates for medical instruments. These assays were developed by adapting a method previously used to test the decontamination of medical devices from contaminations of infectious prion protein (for details see [11]). In brief: Two stainless steel wire grids (100 × 5 mm; DIN 1.4301, Spörl) each were contaminated with 20% (w/v) brain tissue homogenates from patients with AD or demential α-synuclein aggregation disease (SD) and jointly used for the analysis of the respective decontamination treatment. Grids were air-dried for 2 days at room temperature (RT) and subsequently incubated in the formulations for the time-periods and at the temperatures indicated in Figure 1. After incubation in the specified formulations, some grids were additionally steam sterilized at 134°C for the indicated time periods. Residual protein contaminations were eluted from pairs of jointly coiled up wire grids by boiling in 300 μl double-concentrated electrophoresis loading buffer and analysed by Western blotting. 12% Tris-Glycine gels were used for sodium dodecyl sulfate (SDS) polyacrylamide gel electrophoresis (SDS-PAGE), and after SDS-PAGE the whole gels (including the stacking gels) were blotted onto polyvinylidene difluoride membranes. The blots were incubated in the antibody- and blocking solutions specified in Table 1, and labelled proteins were visualized using CDP-star and Amersham Hyperfilm ECL.

**Table 1:** **Antibodies and blocking reagents for Western blot detection of aggregated human α-synuclein, amyloid-β and tau**

**1** ^**st**^ **Antibody**	**Provider and product code**	**Epitope**	**Dilution**	**Blocking solution**	**2** ^**nd**^ **Antibody**	**Dilution**
LB 509 (monoclonal)	Abcam, Cambridge, UK ab27766	aa 115-122 of α-synuclein	1:5,000	0.03% (w/v) Casein in TBS with 0.05% (w/v) Tween 20	Alkaline-phosphatase-conjugated goat anti-mouse IgG (DAKO)	1:5,000
Phospho-S129 (monoclonal)	Abcam, Cambridge, UK ab51253	α-synuclein phosphorylated at Ser129	1:2,000	3% (w/v) Low-fat milk powder in TBS with 0.05% (w/v) Tween 20	Alkaline-phosphatase-conjugated goat anti-rabbit IgG (DAKO)	1:10,000
Anti-tau (monoclonal)	Merck Millipore, Darmstadt, Germany MAB2239	C-terminal region of tau	1:500	3% (w/v) Low-fat milk powder in TBS with 0.05% (w/v) Tween 20	Alkaline-phosphatase-conjugated goat anti-mouse IgG (DAKO)	1:10,000
AT8 (monoclonal)	Thermo Scientific, Rockford, USA MN1020	Phospho-PHF-tau (Ser202/Thr205)	1:500	0.03% (w/v) Casein in TBS with 0.05% (w/v) Tween 20	Alkaline-phosphatase-conjugated goat anti-mouse IgG (DAKO)	1:10,000
6E10 (monoclonal)	Covance, Emeryville, USA SIG-39320	aa 3-8 of Aβ	1:2,000	5% (w/v) Low-fat choco powder in TBS with 0.05% (w/v) Tween 20^a^	Alkaline-phosphatase-conjugated goat anti-mouse IgG (DAKO)	1:10,000
4G8 (monoclonal)	Covance, Emeryville, USA SIG-39220	aa 17-24 of Aβ	1:1,000	5% (w/v) Low-fat choco powder in TBS with 0.05% (w/v) Tween 20^a^	Alkaline-phosphatase-conjugated goat anti-mouse IgG (DAKO)	1:10,000

^a^Tina Zimmermann, personal communication and diploma thesis (2012, Establishment of the organotypic slice culture assay as a model for neurodegenerative diseases. Free University of Berlin, Berlin, Germany).

In our study we focused on large aggregates of pathological Aβ, tau and α-synuclein. These aggregates were largely retained in the stacking gel (indicated in Figure 1 by vertical brackets) and constituted the molecular species which seemed to be most difficult to wash off from the grids below the threshold of detection (Figure 1a-c, lanes “A. bidest. [100 μl], [5 μl], [1 μl]”). Furthermore, our rationale was based on the general assumption that depletion of such protein aggregates from medical instruments concomitantly reduces potentially harmful seeding effects. Recent findings by Duran-Aniotz and colleagues experimentally confirmed this rationale for Aβ aggregates from human AD brain extracts [12].

## Substantial depletion of amyloid-β-, tau- and α-synuclein aggregates on test carriers by exposure to cleaners and steam sterilization

When we assessed the activity of prion-effective reprocessing procedures against Aβ-, tau and α-synuclein aggregates we found that these were simultaneously reduced up to 100-fold, and below the threshold of detection, by alkaline formulations, applied at RT, such as 1 M NaOH (Figure 1a-c, lanes “NaOH-1 h-RT”) or a mixture of 0.2% SDS and 0.3% NaOH without or with subsequent pre-vacuum steam sterilization (PVSS), respectively, for 5 minutes at 134°C (Figure 1a, lane “SDS/NaOH-10'-RT“ and Figure 1b and c, lanes ”SDS/NaOH-10′-RT + 5′-134°C”).

The same depletion effects were observed for α-synuclein aggregates after exposure to more material-friendly formulations such as a commercially available alkaline cleaner [13] for 10 minutes at 55°C (Figure 1a, lane “Alkaline cleaner-10′-55°C”), or a 7.5% H_2_O_2_ solution containing low concentrations of Cu^2+^ ions [14] for 15 minutes at RT (Figure 1a, lane “H_2_O_2_/CuCl_2_-15′-RT”). Similarly effective depletion of Aβ- and tau aggregates was achieved when treatments with the H_2_O_2_/CuCl_2_ formulation or the alkaline cleaner were followed by PVSS at 134°C for 5 or 18 minutes, respectively (Figure 1b and c, lanes “H_2_O_2_/CuCl_2_-15′-RT + 5′-134°C” and “Alkaline cleaner-10′-55°C + 18′-134°C”). These findings of our pilot study suggest that a simultaneous and quantifiably depletion of pathological Aβ-, tau-and α-synuclein aggregates is basically feasible by using prion-effective formulations in routine procedures for the reprocessing of selected medical devices. Peracetic acid (PAA), a commonly used disinfectant being not effective against prions, failed to achieve a detectable depletion of aggregated Aβ, tau and α-synuclein in our assays (Figure 1a-c, lanes “PAA-1 h-RT”).

## How to deal with transmissible protein seeding in the reprocessing of medical devices?

It is open to discussion whether the findings from transmission studies in animals give reason to include specific preventive measures against pathological amyloid-β-, tau- and α-synuclein aggregates in the routine decontamination of certain medical devices. At the same time, studies specifically pursuing the identification and validation of such measures are still scarce. Therefore, available or newly developed assays should be used to experimentally assess on a broader scale the efficacy of reprocessing procedures for medical devices against AD-, PD- or DLB-associated protein aggregates. Ideally, such assessments would also include measurements of the reduction of seeding activity in vitro and/or in vivo. Broadening the data basis from both transmission and decontamination studies could greatly help to answer the question of how to reprocess medical devices in view of transmissible protein seeding.

## Abbreviations

ab: Antibody
Aβ: Amyloid-β
AD: Alzheimer’s disease
DLB: Dementia with Lewy bodies
PAA: Peracetic acid
PD: Parkinson’s disease
PVSS: Pre-vacuum steam sterilization
PrP: Prion protein
RT: Room temperature
SD: Demential α-synuclein aggregation disease
SDS: Sodium dodecyl sulfate
SDS-PAGE: Sodium dodecyl sulfate polyacrylamide gel electrophoresis
